# Enhancing tourist loyalty through location-based service apps: Exploring the roles of digital literacy, perceived ease of use, perceived autonomy, virtual-content congruency, and tourist engagement

**DOI:** 10.1371/journal.pone.0294244

**Published:** 2024-01-31

**Authors:** Shaowei Xiong, Tong Zhang

**Affiliations:** College of Art Design, The College of Post and Telecommunication of WIT, Wuhan, Hubei, China; The Hong Kong Polytechnic University, HONG KONG

## Abstract

Advanced mobile functions and empowered smartphones have provided tourists with various location-based service apps that reshaped the business model of the tourism sector. Despite their importance to tourists, l-apps still have limitations, such as ignorance of tourist preferences and the mismatch between app introduction and tourist experience, therefore affecting tourist loyalty to destinations. Understanding tourist-oriented factors thus becomes critical for l-app designers and service providers. This study integrates the technology-acceptance model (TAM) into a unique context to examine the roles of digital literacy, perceived ease of use, perceived autonomy, virtual-content congruence, and tourist engagement on tourist loyalty. Our empirical test of a structural equation model based on a randomly recruited 319 customers found that tourists’ digital literacy influences their engagement and perceived ease of use, which mediates the relationship between digital literacy and engagement; tourists’ perceived autonomy influences their engagement. Moreover, we found the moderating role of information-experience congruency between digital literacy, perceived ease of use, and perceived autonomy and tourist engagement, thus contributing to the boundary conditions of the TAM model. Finally, tourist engagement contributes to tourist loyalty. The study contributes to the integration of the technology acceptance model with a tourist orientation. The findings also offer meaningful, practical implications and recommendations on l-app design to stakeholders of tourist destinations.

## Introduction

The development of the 5G mobile communication technology, and mobile functions (e.g., cameras, sensors, & gyroscopes) has empowered smartphones to be responsive to information retrieved directly from users’ physical surroundings [1]. In China, around 932 million users now rely on mobile phones to access the Internet [2]. These users can achieve various online interactions and services thanks to the wireless data networks that enable the development and launch of software such as location-based service apps (l-apps) [3]. Location-based service apps refer to mobile applications that use geolocation technology to provide users with personalized and location-specific recommendations for products or services [4].

The development mentioned above further reshaped the business models in the service sector, which requires firms to integrate their services into mobile devices [5, 6]. L-apps allow users to discover and access nearby businesses, events, promotions, and deals based on their current location, preferences, and search history [7]. The prevalent l-apps include 1) travel and tourism apps (e.g., apps providing information on nearby tourist attractions, restaurants, hotels, & transportation services) that feature user reviews, ratings, and photos to help tourists make informed decisions [8], 2) food and drink apps that suggest nearby restaurants, bars, cafes, and food delivery services based on the user’s location, cuisine preferences, and budget [9], 3) retail and shopping apps that provide location-based promotions, discounts, and deals for nearby stores, malls, and online retailers [10], 4) entertainment and events apps that inform users about nearby concerts, movies, festivals, sports events, and other entertainment options [11], and 5) transportation and mobility apps that help users navigate and access various transportation options, such as taxis, ride-sharing services, public transit, and bike-sharing programs [12].

While providing conveniences to local consumers, l-apps can be especially important for tourists, who seek personalized, real-time information that enhances their travel experiences [13]. In particular, tourists often have limited time in a destination, and l-apps can help them make the most of their visit by providing customized information based on their interests and preferences [8]. As such, tourists can use l-apps to find popular local spots, recommended restaurants, and events happening in real time. L-apps can also help tourists navigate unfamiliar places, as they can receive step-by-step directions to their desired destination [14]. In addition to providing convenience and personalization, l-apps can also enhance the safety and security of tourists [13]. For example, tourists can use l-apps to locate emergency services, such as hospitals or police stations, in an emergency [15]. L-apps can also help tourists avoid unsafe or undesirable areas by providing real-time information on safety conditions or potential hazards in the area. In other words, technologies such as l-apps can provide tourists with proactive traveling experience, enabling them to become decision-makers in tourism experiences [16].

Regardless of these conveniences, tourists still experience several issues when using l-apps. First, while tourism apps can recommend places and activities based on a user’s location, they often fail to consider tourists’ individual preferences and interests [17]. Second, tourism apps often fail to provide tourists with emotional connections to the destination [18]. For instance, those apps may inform tourists about a landmark’s history or data about a city, but they do not capture the emotional experience of being there. Also, tourism apps are unable to facilitate interaction with local residents [19], although local interaction can be a valuable and enriching experience for many tourists. Interactions with locals can provide a deeper understanding of the local culture, customs, and traditions [19]. Third, while some tourism apps have functions for virtual tours, they are not powerful enough for fully immersive experiences [20]. While a tourist appreciates the convenience of an audio guide in a museum, he or she is unable to be fully immersed in the exhibit. Fourth, tourism apps may provide information on popular attractions and activities, but they are unable to provide help when tourists experience unexpected situations such as a missed flight or a medical emergency [21]. As a result, tourists may rely not only on tourism apps but also other l-apps (e.g., local supermarkets, restaurants, food deliveries, medical services, local bus/metro apps) that are not available in tourism apps.

Another essential gap in the l-app study has been the congruency between the information provided by l-apps and the actual experience felt by tourists. Indeed, 1-apps often use professional photographs and videos that are taken in ideal conditions, with perfect lighting and angles [22]. These images are carefully selected to showcase the best features of a particular attraction or destination. However, when tourists visit a site in person, they may encounter a different reality. They may see crowds of people, long lines, and busy streets, which are not always visible in the carefully curated images presented in the app [23]. Another difference between an l-app and a real-life experience is that apps can recommend services that meet tourists’ various needs. Those recommendations are not always up-to-date or reliable, and the actual experience of visiting a location can be quite different from what is portrayed in the app [24]. Moreover, tourist apps cannot capture the sensory experiences of being in a particular location, such as the smell of the air, the taste of the food, or the sounds of the surroundings. These sensory stimuli are important in creating an authentic experience of a place and can only be fully appreciated by visiting in person [25]. In summary, l-apps provide a helpful overview of a location, but they cannot fully replicate the experience of being there in person. Tourists should use these apps as a starting point for their travel planning and research, but they should also be prepared for unexpected challenges and experiences that may arise during their travels. This study recognizes this issue and examines the important role of information-experience congruency in the

Several studies [26, 27] have shed light on the connection between tourism app design and tourists’ engagement in a tourist destination. Tourist engagement refers to the emotional and mental state that arises when tourists actively participate in interactive and collaborative experiences with a specific focus, such as people, attractions, activities, or encounters, within the context of their travel experiences [28]. Former studies have recognized tourist engagement towards a tourism destination as an important predictor of tourist loyalty and revisit intentions [29–31], with some scholars highlighting the important role of tourism apps on tourist engagement [32]. Not much has been written about the role of l-apps (including but not limited to tourism apps) in helping tourist to form perceived experiential values that further influence their engagement and loyalty. In particular, it is worth investigating the mechanisms where l-apps influence tourist engagement in a tourism-related context rather than a general consumption or retailing context. Such an exploration seems important as it can help tourism app developers improve product quality.

A prevalent theoretical framework to understand users’ experience in digital services is the technology acceptance model (TAM), which explains how users’ cognitive features affect their experience and acceptance of digital-mediated services such as tourism apps [33]. However, several scholars [34–36] further suggest that the TAM model should be integrated with a user’s experience perspective in explaining consumers’ experience when using mobile apps. For instance [36], calls for more research to explore the role of users’ perceived ease of use, which may depend on users’ proficiency in using l-apps (i.e., digital literacy); likewise [37], suggest that tourists’ motivation to obtain autonomy during travel should be considered. Therefore, this study integrates users’ digital literacy and their perception of the congruency between digital app presentations and their actual experiences (i.e., information-experience congruency) in tourist destinations into the TAM model, with the purpose of examining whether and how tourists’ digital literacy, perceived ease of use, perceived autonomy during app usage, and the information-experience congruency collectively influence their engagement, which further affects their loyalty toward the tourist destination.

## Theoretical background and hypothesis development

### Tourist engagement from l-apps

L-app developers often seek to design various functions that help service providers, such as tourist destinations to create atmospheres and services that create pleasant experiences and improve tourist engagement. Engagement refers to an individual’s favorable response to technology-mediated services [38]. According to [39], consumers’ engagement involves their cognitive, emotional, and interactive processes. In other words, isolating tourists’ psychological activities from their behavioral activities in a specific tourist destination can not effectively reflect tourist engagement. Several scholars [40] recommend context-specific examinations for consumer engagement due to its interactive and context-defined nature.

Tourists, especially first-time visitors, are often unfamiliar with the physical environment in a tourist destination. Before and during their traveling, tourists often rely on l-apps for critical information related to their travel and develop expectations towards the services and experiences in the tourism destination [41]. Therefore, l-apps developers often integrate visual and textual information to improve tourist engagement. So far, not much has been written about how tourists’ cognitive and online and offline interactive activities affect their engagement in a tourist destination.

### Contextualized technology acceptance model

Davis’s technology acceptance model (TAM) has been adapted in tourism studies to examine users’ acceptance of technological services (e.g., apps & robots) [40, 42, 43]. The TAM suggests that the designed features could affect consumers’ perceived usefulness and ease of use, influencing their attitudes and intention in using and purchasing a technology [44]. In a tourism context, the role of perceived usefulness (convenience, efficiency, & cost-savings) has been widely examined and confirmed [45, 46]. However, we argue that tourists’ perceived ease of use should not be taken for granted. While designers may provide useful functions, tourists have varying levels of proficiency in using digital apps; which could affect their perceptions. Moreover, tourists’ varied needs suggest that whether l-apps could enable them to maximize the various benefits of l-apps according to their travel plan could also affect their attitudes. Tourists’ favorable attitudes towards a destination when using l-apps (i.e., tourist engagement) may also depend on the consistency between the promised benefits and the actual experience they have when buying tourist services. Drawing on the above considerations, this study proposes a modified TAM model to examine the antecedents of tourist engagement and the app-mediated impact on tourist attitudes towards tourism destinations.

### Digital literacy and engagement

When searching for information related to a tourist destination, l-app users can be restrained by their digital literacy, i.e., abilities to access, evaluate, and utilize digital information to meet various needs [47]. Digital literacy includes the essential searching and interactive skills required for app users to navigate the digital environment [48]. While l-app designers include various functions based on user habits to provide interactive apps for users to obtain the information that meets their needs, users’ digital literacy could lead to different search results, thus, different degrees of satisfaction. The documented examples of users’ digital literacy include 1) app navigation, i.e., abilities to understand and use l-app functions; 2) understanding the underlying logic and technology of a specific l-app to troubleshoot problems and reduce frustration when using the app; 3) confidence in the information found on the l-app; and 4) integrate the l-apps with other facilities (e.g., self-help services) to improve the overall satisfaction of the experience [49, 50]. Therefore, it can be hypothesized that digital literacy positively affects tourists’ engagement and the following can be hypothesized:

H1: A tourist’s digital literacy for l-apps is positively associated with his or her engagement in a tourist destination.

### Perceived ease of use and engagement

As an important antecedent to user attitude, perceived ease of use suggests whether a consumer believes that using a particular app would require little effort [51, 52]. The role of perceived ease of use in consumers’ intention to l-apps has been verified in several studies [53, 54]. In the tourism scenario, user-friendly apps can help tourists effectively find the required information by reducing the psychological cost when searching for the information [55]. When visiting a new place, tourists can be overwhelmed by the information by different l-apps that feature different functions [56]. When tourists perceive the limited efforts required to find the relevant information and, more importantly, then verify the quality of the recommended information through an actual experience, they are more likely to develop engagement. However, as mentioned above, perceived ease of use depends on the digital literacy of tourists. In particular, tourists who usually use l-apps on various occasions are more likely to develop the skills and experience that enable them to develop perceived ease of use, which further influences their engagement. As a result, the following can be hypothesized:

H2: A tourist’s perceived ease of use for apps is positively associated with his or her engagement in a tourist destination.

H2a: A tourist’s perceived ease of use positively mediates the relationship between digital literacy and his or her engagement in a tourist destination.

### Perceived autonomy and engagement

Despite the convenience provided by l-apps, tourists often hope to appropriate and personalize the tourism experience [57]. This involves tourists’ efforts to acquire relevant information and develop knowledge about a specific tourist destination, and they rely on the information to feel comfortable throughout the traveling process [58]. To develop engagement, tourists need to feel free to initiate their own actions, with the support of l-apps, in a tourist destination, i.e., autonomy. For instance, tourists may bear personalized goals and expectations before the tour and then proactively search and filter information from advertisements, recommended products, and services, and then decide which service to select [59]. When l-apps are designed in a way that allows tourists to feel autonomy, those tourists are able to invest sufficient effort in the experience and focus on appreciating the specific services of the destination, which may improve their satisfaction. In other words, l-apps that are designed in ways that allow tourists to autonomously enhance their sense of control could improve their engagement. As a result, the following hypothesis could be predicted:

H3: A tourist’s perceived autonomy is positively associated with his or her engagement in a tourist destination.

### Moderating effect of information-experience congruency

While l-apps can provide important information through pictures and videos to enhance tourists’ engagement, the congruency of such information to the actual services that tourists receive is critical. According to [60], congruency refers to the match between the described feature and the experienced feature of a stimulus. Audio and digital technologies now allow tourist destinations and affiliated service providers to provide various visual cues to increase tourists’ chances of visiting [61]. Indeed, a visually appealing tourist destination with convenient transportation, cultural relics, and good services can strengthen tourists’ emotive expectations. However, tourist engagement in the tourist destination depends not only on tourists’ digital literacy to find the popular scenic spots, restaurants, and activities at the lowest prices through user-friendly apps and plan trips according to their preferences; but also on the congruency between the contents (e.g., prices, promised service quality, and overall experience) and the visual cues on l-apps. For instance, tourists attracted by the appealing pictures and high ratings of a popular restaurant in a tourist city may feel disappointed if the food and service quality is lower due to peak season. Previous studies [62, 63] have confirmed that congruency can have a positive impact on consumers’ emotional reactions toward the tourism experience and their engagement. As a result, the following hypothesis can be developed:

H4a: Information-experience congruency positively moderates the relationship between a tourist’s digital literacy for l-app and his or her tourist engagement when visiting a tourist destination.

H4b: Information-experience congruency positively moderates the relationship between a tourist’s perceived ease of use for l-app and his or her tourist engagement when visiting a tourist destination.

H4c: Information-experience congruency positively moderates the relationship between a tourist’s perceived autonomy from l-app and his or her tourist engagement when visiting a tourist destination.

### Tourist engagement and tourist loyalty

The positive impacts (e.g., satisfaction, trust, & loyalty) of tourist engagement has been confirmed in different contexts [39]. Tourist engagement involves the tourist’s cognitive and emotional processes that are responsible for their satisfaction. While l-apps could allow tourists to appreciate the various scenic spots and services on their self-designed tourist routes, the actual satisfaction towards the tourist destination involves several components, such as tourists’ participation in local events, interactions with service providers and local residents, and felt a connectedness with specific elements or actors in the destination [64]. These can be achieved through tourist engagement, which could enhance the tourists’ intentions to recommend the destination to others and revisit the destination in the future [65]. Hence, we predict:

H5: Tourist engagement is positively associated with tourist loyalty towards a tourist destination.

**Fig 1:**
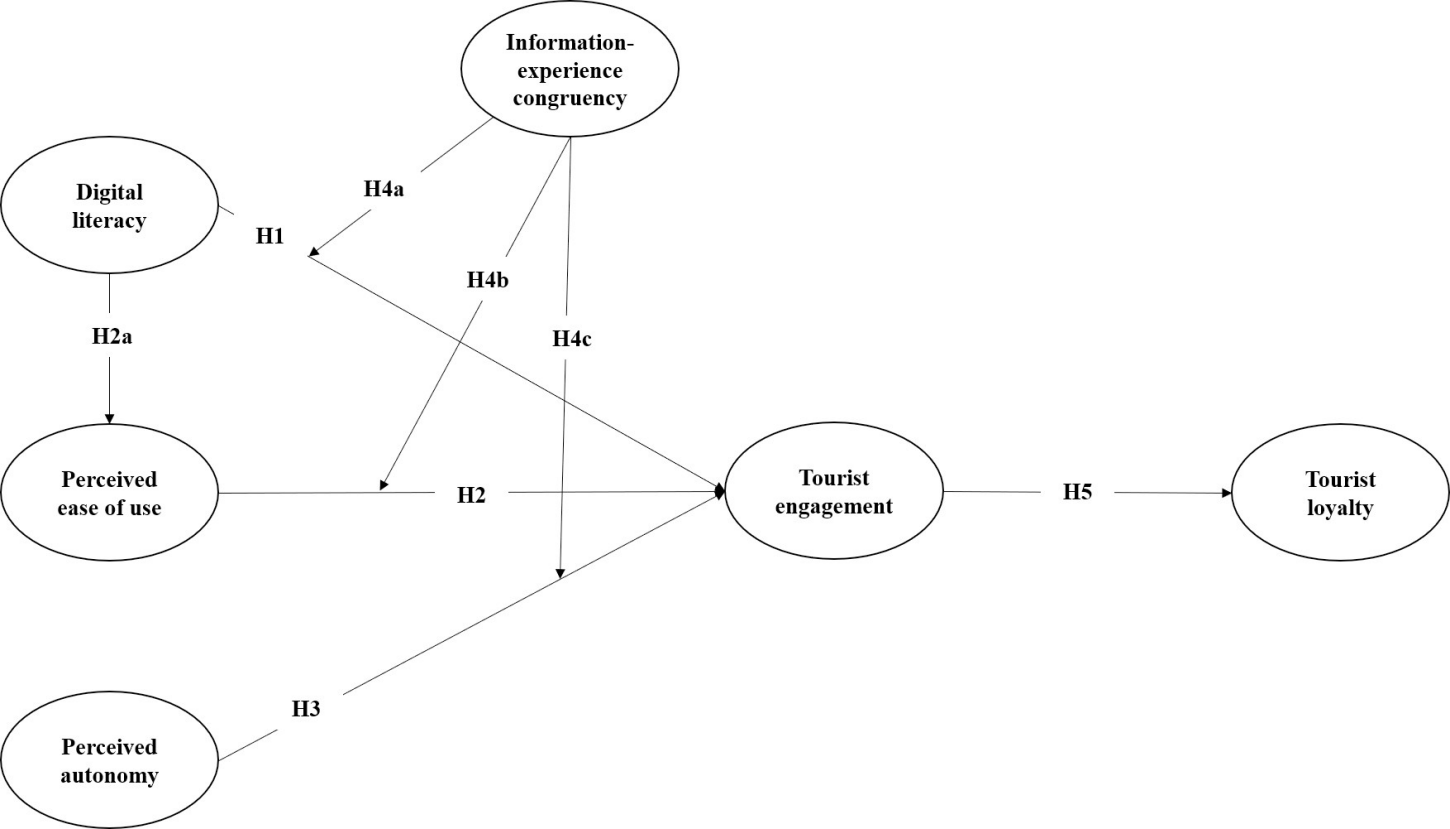
Conceptual model.

The above hypotheses constitute the conceptual model of this study (see Fig 1.)

## Methods

### Sampling and data collection

This study examined the hypothesized relationships by surveying customers in China. Before data collection, we obtained ethical approval from Research Ethics Committee of The College of Post and Telecommunication of WIT, as well as informed consents from participants. We randomly invited 400 customers of 71384 users of a tourist center in a central Chinese city through the Internet on Feb, 1^st^, 2023. The center relies heavily on a location-based consumption app (Dazhongdianping.com) to attract tourists. The authors had no access to information that could identify individual participants during or after data collection. We sent out the survey via ’WENJUANXING’ (a questionnaire survey platform widely used in China) on the Feb, 3^rd^, 2023. In the survey webpage, we first introduced the objective of this study. The self-reported scale was performed on six main variables (digital literacy, perceived ease of use, perceived autonomy, information-experience congruency, tourist engagement, and tourist loyalty) in order to examine the mechanism underlying the tourist loyalty of customers. We received 346 responses by Feb, 28, 2023; after excluding 17 invalid questionnaires, the sample for analysis consisted of 319 responses, or 92.2% of the total. 52.4% of the respondents were male (N = 167), and 47.6% of the respondents were female (N = 152). 21.9% of the respondents were 36–40 years old (N = 70), 20.7% of the respondents were 31–35 years old (N = 66), 20.1% of the respondents were 26–30 years old (N = 64), 19.7% of the respondents were 41 years old and above (N = 63), 17.6% of the respondents were 20–25 years old (N = 56). 81 respondents (25.4%) reported an income range between 7501 and 9500 yuan, 67 respondents (21%) reported an income range between 9501 yuan and above, 66 respondents (20.7%) reported an income range between 3501 and 5500 yuan, 65 respondents (20.4%) reported an income range between 5501 and 7500 yuan, 40 respondents (12.5%) reported an income range between 3500 yuan and below. Table 1 presents the demographic information of those respondents.

**Table 1:** Demographic information.

Demographics		Frequency	Percentage
**Gender**	Male	167	52.4%
	Female	152	47.6%
**Age**	20–25 years old	56	17.6%
	26–30 years old	64	20.1%
	31–35 years old	66	20.7%
	36–40 years old	70	21.9%
	41 years old and above	63	19.7%
**Income**	3500 yuan and below	40	12.5%
	3501–5500 yuan	66	20.7%
	5501–7500 yuan	65	20.4%
	7501–9500 yuan	81	25.4%
	9501 yuan and above	67	21.0%

### Measures

The questionnaire for this study included six constructs: digital literacy, perceived ease of use, perceived autonomy, information-experience congruency, tourist engagement, and tourist loyalty. We translated each item into Chinese because our respondents are Chinese [66]. We invited bilingual researchers in consumer behavior to translate the items into Chinese and then into English. A 5-point Likert scale was used for each measurement item.

#### Digital literacy

Digital literacy was measured by a 7-item scale adapted from Rodríguez-de-Dios, Igartua [67]. Sample items include ‘Download/save a destination picture I found online’, ‘Download tourism information I found online’, and ‘I don’t like apps on my phone because I find them difficult to use’. Cronbach’s Alpha = 0.730.

#### Perceived ease of use

Perceived ease of use was measured by a 4-item scale adapted from Kim and Lee [68]. Sample items include ‘Ease of navigation’, ‘Ease of online reservation’, and ‘Ease of online cancellation’. Cronbach’s Alpha = 0.820.

#### Perceived autonomy

Perceived autonomy was measured by a 3-item scale adapted from Lunardo and Ponsignon [69]. Sample items include ‘I would be (was) able to choose where to go to’, ‘I could freely choose what area I want to explore’, and ‘I would have (had) absolute full control over where I could go’. Cronbach’s Alpha = 0.951.

#### Information-experience congruency

Information-experience congruency was measured by a 4-item scale adapted from O’Cass and Grace [70]. Sample items include ‘Consistent with experience’, ‘People similar use App’, and ‘Reflects what I search’. Cronbach’s Alpha = 0.790.

#### Tourist engagement

Tourist engagement was measured by a 7-item (two dimensions: immersed involvement and novelty seeking) scale adapted from Huang and Choi [71]. Sample items include ‘I was completely involved in all of the activities I participated in’, ‘I was enthusiastic each day to make this trip memorable’, and ‘I spontaneously engaged in unexpected activities’. Cronbach’s Alpha of immersed involvement = 0.860, Cronbach’s Alpha of novelty seeking = 0.839.

#### Tourist loyalty

Tourist loyalty was measured by a 6-item scale adapted from Loureiro and González [72]. Sample items include ‘I will speak well about this place to other people’, ‘I will encourage my friends and relatives to visit this place’, and ‘I would come continually even if the ticket price increases’. Cronbach’s Alpha = 0.968.

### Data analysis

A measurement model was first established via confirmatory factor analysis (CFA), using AMOS 24.0, and then the reliability analysis and correlation analysis were conducted by using SPSS 25.0. In order to avoid the problem of common method bias, we statistically tested the potential influence of common method bias using Harman’s single-factor test to minimize potential common method bias. We adopted bootstrapping to test the indirect effect. We used Process Macro Model 1 was used to test the moderating effects. The simple slopes of the moderating effect were plotted according to the suggestion by [73].

## Results

### Common method bias

Harman’s single-factor test was utilized to examine the problem of common method bias. We identified six factors with eigenvalues greater than 1, with the first factor explaining less than 40% of the variance (35.75% of 76.02%). Therefore, our findings provided no serious indications of common method variance.

### Measurement model

We tested the convergent validity and discriminant validity of the proposed model. The purpose is to confirm the adequacy of the constructs used in the model.

#### Convergent validity

The CFA results indicated that the data had a good fit for the measurement model. CMIN/DF = 1.421, root mean square error of approximation (RMSEA) = 0.036, root mean square residual (RMR) = 0.048, and comparative fit index (CFI) = 0.975. For evaluating convergent validity, [74] to test composite (construct) reliability (CR), item reliability, and average variance extracted (AVE). We conducted the reliability test before assessing validity [75]. We examined assess internal consistency among the constructs that stipulated the construct reliability through Cronbach’s alpha (α). Table 2 shows that all construct’s alpha values ranged from 0.826 to 0.943 exposing high construct reliability.

**Table 2:** Results of validity and reliability.

Constructs		Items	Factor loadings	CR	AVE	Cronbach’s Alpha
**Digital literacy**		DL1	0.787	0.943	0.705	0.943
		DL2	0.891			
		DL3	0.855			
		DL4	0.813			
		DL5	0.832			
		DL6	0.810			
		DL7	0.882			
**Perceived ease of use**		PEOU1	0.693	0.885	0.661	0.883
		PEOU2	0.892			
		PEOU3	0.770			
		PEOU4	0.881			
**Perceived autonomy**		PA1	0.743	0.827	0.614	0.826
		PA2	0.807			
		PA3	0.799			
**Tourist engagement**	**Immersed involvement**	II1	0.846	0.859	0.670	0.858
		II2	0.830			
		II3	0.778			
	**Novelty seeking**	NS1	0.848	0.925	0.756	0.919
		NS2	0.813			
		NS3	0.903			
		NS4	0.910			
**Tourist loyalty**		TL1	0.848	0.926	0.676	0.925
		TL2	0.820			
		TL3	0.721			
		TL4	0.790			
		TL5	0.910			
		TL6	0.833			
**Information-experience congruency**		IEC1	0.770	0.884	0.656	0.883
		IEC2	0.793			
		IEC3	0.818			
		IEC4	0.855			

We also checked factor loading to ensure item reliability. [75] recommended that as a general principle, 0.5 or higher value represents a significant measurement. In Table 2, the loadings ranged from 0.693 to 0.910, which demonstrates higher item reliability. According to [74], the AVE value of each construct should be higher than 0.5, and Table 2 shows that the each construct met this requirement. Therefore, we had a satisfactory convergent validity.

#### Discriminant validity

We examined discriminant validity through the squared correlations between two separate weights in either construct; expecting that the value to be lower than the variance shared by the measures of a construct [76]. Table 3 shows the result of the discriminant validity test. The square roots of AVEs were greater than their correlation coefficients with other factors that strongly support the discriminant validity.

**Table 3:** Results of correlation and discrimination analysis.

Constructs	Mean	SD	1	2	3	4	5	6	7	8	9
**1. Gender**	1.48	0.50	--								
**2. Age**	3.06	1.38	-.121*	--							
**3. Income**	3.22	1.33	0.01	0.061	--						
**4. Digital literacy**	3.50	1.06	0.016	-0.001	-0.013	0.839					
**5. Perceived ease of use**	3.63	0.85	-0.017	-0.006	-0.009	.534**	0.813				
**6. Perceived autonomy**	3.82	1.00	0.001	-0.041	-0.018	.555**	.474**	0.784			
**7. Tourist engagement**	3.79	0.83	0.035	-0.036	-0.033	.486**	.423**	.390**	0.825		
**8. Information-experience congruency**	3.68	1.03	0.04	0.032	-0.054	.158**	.129*	0.063	.232**	0.81	
**9. Tourist loyalty**	3.72	0.93	-0.056	0.078	0.007	.401**	.352**	.358**	.355**	.294**	0.822

*, p<0.05

**, p<0.01. The diagonal values are square roots of AVEs.

### Hypothesis testing

#### Direct effects and indirect effects

**Fig 2:**
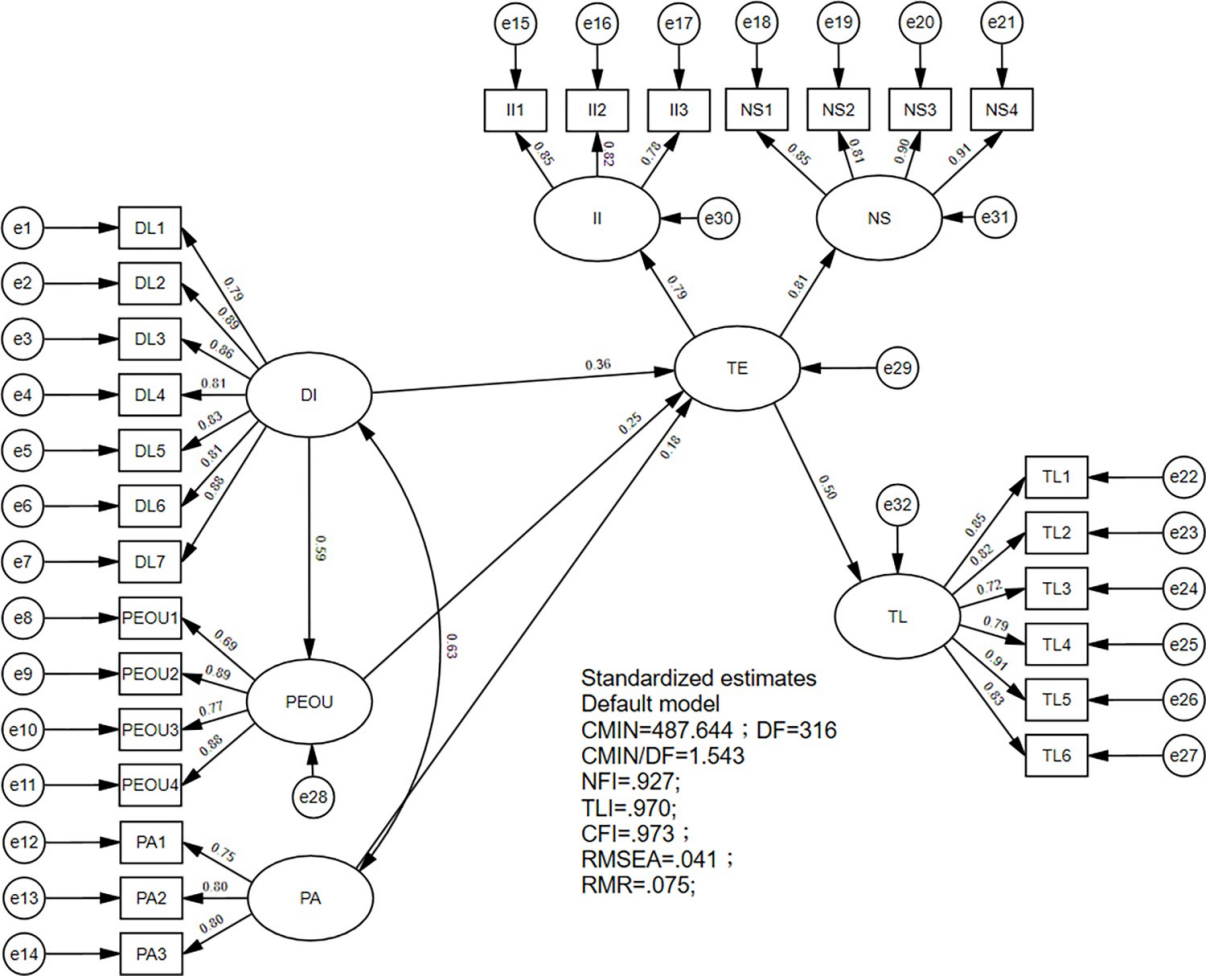
Structural model.

This study has constructed a structural model for examining the correspondence among the variables. The structural equation model (Fig 2) results indicate that the data has a good fit for the structural model (CMIN/DF = 1.543, root mean square error of approximation (RMSEA) = 0.041, root mean square residual (RMR) = 0.075, and comparative fit index (CFI) = 0.973). Table 4 demonstrates that DL has a significant positive effect (β = 0.585, p<0.001) on PEOU; DL has a significant positive effect (β = 0.363, p<0.001) on TE; PEOU has a significant positive effect (β = 0.248, p<0.001) on TE; PA has a significant positive effect (β = 0.178, p = 0.03) on TE; TE has a significant positive effect (β = 0.497, p<0.001) on TL. Thus, H1, H2, H3, and H5 are supported. The indirect effect is analysed, and the result is presented in Table 5. The indirect effect of DL on TE via PEOU is 0.145, and bootstrapped 95% CI did not include zero (0.070, 0.247). Thus, H2a is supported.

**Table 4:** Results of direct effects.

Path			STD. Estimate	S.E.	C.R.	P	Result
PEOU	<---	DL	0.585	0.042	8.797	***	Supported
TE	<---	DL	0.363	0.062	3.889	***	Supported
TE	<---	PEOU	0.248	0.079	3.305	***	Supported
TE	<---	PA	0.178	0.062	2.168	0.030	Supported
TL	<---	TE	0.497	0.096	6.980	***	Supported

***, p<0.001; DL, digital literacy; PEOU, perceived ease of use; PA, perceived autonomy; TE, tourist engagement; TL, tourist loyalty

**Table 5:** Result of the indirect effect.

Indirect effect	Estimate	S.E.	Lower	Upper
DL-PEOU-TE	0.145	0.044	0.070	0.247

DL, digital literacy; PEOU, perceived ease of use; TE, tourist engagement

#### Moderating effect

**Fig 3:**
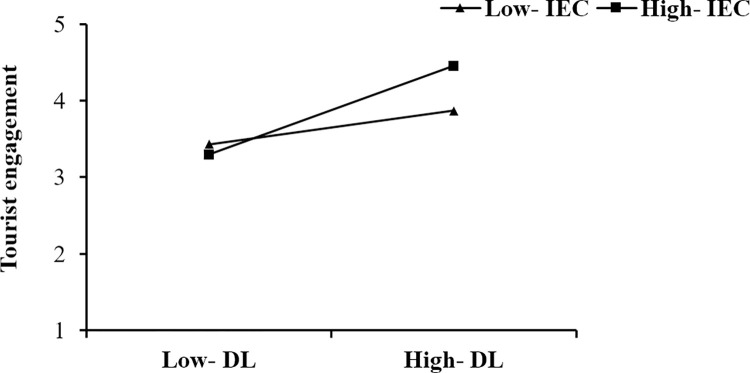
The moderating effect of IEC on the relationship between DI and TE.

**Fig 4:**
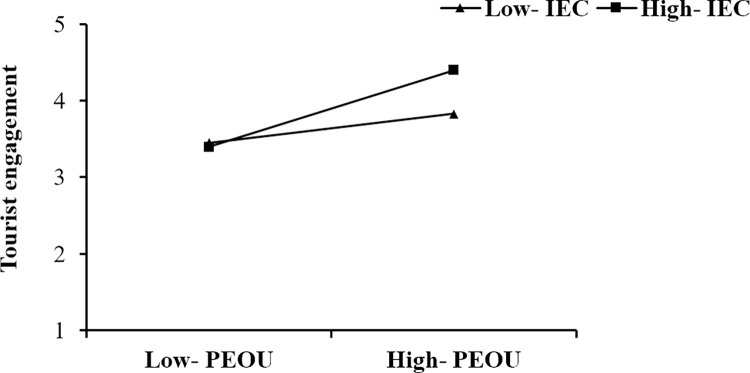
The moderating effect of IEC on the relationship between PEOU and TE.

**Fig 5:**
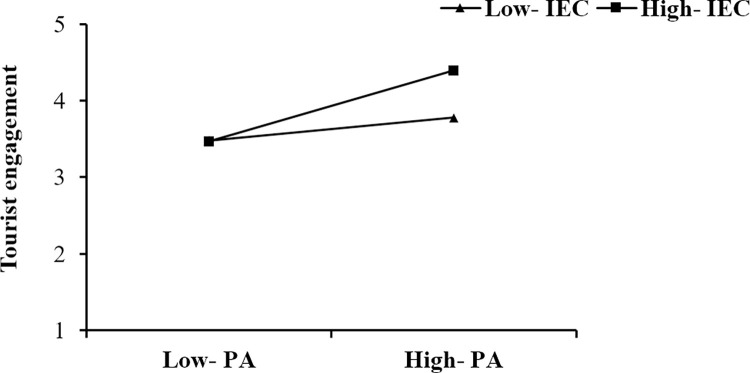
The moderating effect of IEC on the relationship between PA and TE.

The moderating effects are analysed, and the results are presented in Table 6. As shown, the moderating effect of IEC on the relationship between DI and TE is significant, that is, when IEC is at a high level (Mean +1SD), the relationship between DI and TE is stronger than it is at a low level (Mean -1SD) (a simple slopes test presented in Fig 3). The moderating effect of IEC on the relationship between PEOU and TE is significant, that is, when IEC is at a high level (Mean +1SD), the relationship between PEOU and TE is stronger than it is at a low level (Mean -1SD) (a simple slopes test presented in Fig 4). The moderating effect of IEC on the relationship between PA and TE is significant, that is, when IEC is at a high level (Mean +1SD), the relationship between PA and TE is stronger than it is at a low level (Mean -1SD) (a simple slopes test presented in Fig 5). Thus, H4a, H4b, and H4c are supported.

**Table 6:** Result of moderating effects.

Variable	IEC	Effect	se	t	LLCI	ULCI
**DI**	Mean-1SD	0.208	0.053	3.954	0.105	0.312
	Mean+1SD	0.549	0.058	9.436	0.434	0.663
**PEOU**	Mean-1SD	0.222	0.068	3.267	0.088	0.355
	Mean+1SD	0.587	0.074	7.908	0.441	0.733
**PA**	Mean-1SD	0.149	0.064	2.322	0.023	0.275
	Mean+1SD	0.464	0.061	7.663	0.345	0.583

DL, digital literacy; PEOU, perceived ease of use; PA, perceived autonomy; TE, tourist engagement; IEC, information-experience congruency

## Discussion and conclusions

Tourists with limited-time often rely on l-apps to optimize the tourism experience. L-app designers are expected to address various issues, such as recommendations ignoring tourists’ preferences, limited emotional connections, and immersive experiences, together with disparate information between l-app capture and tourist experience. Given these issues, this study aims to investigate tourists’ perceptions of l-apps to determine whether and how tourists’ digital literacy, perceived ease of use, perceived autonomy during app usage, and the information-experience congruency could collectively influence tourist engagement, which further affects their loyalty toward the tourist destination. Our results are generalizable to similar l-apps used by tourists in China.

In particular, our results suggest that tourists’ digital literacy has a positive impact on their engagement. This finding concurs with previous studies that have highlighted the importance of digital literacy in enhancing tourists’ engagement [47, 48]. It is widely acknowledged that tourists’ ability to effectively use digital tools is crucial for their engagement [49]. These findings further reinforce the idea that tourists’ digital literacy plays a significant role in shaping their level of engagement with tourism experiences. We also confirmed the positive influence of tourists’ perceived ease of use of l-apps on tourist engagement. Studies [56] have consistently emphasized the significance of user-friendly interfaces and intuitive design in enhancing user engagement. This finding suggests that tourists are more likely to engage with l-apps when they perceive them as easy to use, underscoring the importance of user experience design in driving engagement. Moreover, we confirmed the mediating role of perceived ease of use between digital literacy and tourist engagement. This concurs with [53] that has explored how perceived ease of use mediates in the relationships involving technology adoption and usage. Our results further suggest that l-app designers should not only keep those apps technologically accessible but also easy to navigate and use. A seamless user experience can contribute to higher levels of engagement among digitally literate tourists. Additionally, we confirmed the positive impact of tourists’ perceived autonomy in destinations on their engagement: This result aligns with previous literature [28] on motivation and engagement in tourism contexts. Autonomy, as a psychological need, has been recognized as an essential factor in fostering engagement and satisfaction [59]. This finding underscores the importance of providing tourists with opportunities to make choices and exert control over their experiences within tourist destinations. Enabling autonomy can contribute to higher levels of engagement and satisfaction among tourists.

We also identified the moderating role of information-experience congruency. This finding extends previous studies [44] that only assume the positive role of l-app features. In fact, information-experience congruency is very important for tourists whose digital literacy only allows them to rely on the immediately available information on l-apps. These tourists often assume the l-app to provide accurate and reliable information, although such information can be different due to tourism seasons. Second, information-experience congruency enhances the impact of perceived ease of use on tourist engagement, and information-experience congruency enhances the impact of perceived autonomy on tourist engagement since congruency allows them to enjoy the tourism experience according to plans. These findings further proved the boundary conditions of digital literacy, perceived ease of use, and perceived autonomy on tourist engagement.

Finally, our finding on the positive impact of tourist engagement on tourist loyalty is consistent with the existing body of research [39]. Engaged tourists are more likely to have positive experiences, develop emotional connections with the destination, and exhibit repeat visitation and word-of-mouth recommendations. This finding reinforces the notion that engaging tourists throughout their journey can lead to long-term loyalty and advocacy, which are crucial for the sustainable success of tourism destinations and businesses.

### Theoretical implications

We contribute to the theoretical understanding of tourist engagement by shedding light on the role of digital literacy, perceived ease of use, perceived autonomy, and their interplay in shaping engagement levels. Specifically, we expand the understanding of the role of technology on tourist engagement by unraveling the mechanisms under which tourist engagement in a destination is influenced not only by experience at tourist sites but also by the overall consumption experience. By highlighting the mediating effect of perceived ease of use, the study extends the theoretical framework of technology adoption [33] and user experience in the tourism context [48]. We provide empirical support to the theoretical foundation that engagement is influenced by various factors, including individuals’ digital literacy, perceived ease of use, and perceived autonomy.

### Practical implications

We also make two important practical implications. First, this study emphasizes the importance of considering tourists’ digital literacy when designing tourism apps and digital platforms. App developers and tourism organizations could consider providing tutorials with clear instructions to enhance tourists’ digital literacy and overall usability. Second, the study highlights the significance of fostering perceived autonomy within tourist destinations. Tourism stakeholders (tourist sites & local service providers) should strive to create opportunities for tourists to make choices and personalize their experiences, rely on l-apps to develop innovative services to enrich tourists’ experiences [16], and provide authentic information that matches tourists’ actual experiences, thereby improving tourist engagement.

### Limitations and suggestions for future studies

We acknowledge a few limitations, which provide a comprehensive understanding of its scope and implications. First, the research focused on a specific tourist population and may not capture the diversity of tourists across different destinations and contexts. Future studies should consider including a more diverse sample to ensure the generalizability of the findings. Second, we used self-report measures, which may generate potential biases such as social desirability and memory recall. To enhance the validity of the results, future research could incorporate observational data or objective measures to complement self-reported data. Also, future studies could adopt qualitative research methods, such as interviews or focus groups, to gain in-depth insights into tourists’ experiences and perceptions regarding digital literacy, ease of use, autonomy, engagement, and loyalty. This qualitative data can complement the quantitative findings and provide a richer understanding of the underlying mechanisms and dynamics at play.

Third, the study examined the impact of digital literacy, perceived ease of use, perceived autonomy, and tourist engagement on tourist loyalty, but it did not explore other potential factors that may influence loyalty, such as satisfaction or perceived value. Future studies could consider incorporating these additional variables for a fuller understanding of the loyalty-building process. Lastly, the study employed a cross-sectional design, which limits the ability to establish causality or temporal relationships. Longitudinal studies or experimental designs could be conducted in the future to examine the dynamic nature of the relationships between the variables over time and to further explore causal relationships.

## Supporting information

S1 Checklist *PLOS ONE* clinical studies checklist. (DOCX)

S2 Checklist STROBE statement—checklist of items that should be included in reports of observational studies. (DOCX)

S1 File (PDF)

S1 Dataset (XLSX)
