# Supplementary material for: Enhancing tourist loyalty through location-based service apps: Exploring the roles of digital literacy, perceived ease of use, perceived autonomy, virtual-content congruency, and tourist engagement
Source: PLoS One. 2024 Jan 31;19(1):e0294244. doi: 10.1371/journal.pone.0294244 (PMC10830003; doi:10.1371/journal.pone.0294244)
Supplement: S2 Checklist — (DOCX) [file pone.0294244.s002.docx]

STROBE Statement—checklist of items that should be included in reports of observational studies

|  | Item No. | Recommendation | Page  No. | Relevant text from manuscript |
| --- | --- | --- | --- | --- |
| **Title and abstract** | 1 | (*a*) Indicate the study’s design with a commonly used term in the title or the abstract | 1 | Our empirical test of a structural equation model based on a randomly recruited 319 customers |
|  |  | (*b*) Provide in the abstract an informative and balanced summary of what was done and what was found | 1 | tourists’ digital literacy influences their engagement and perceived ease of use, which mediates the relationship between digital literacy and engagement; tourists’ perceived autonomy influences their engagement |
| Introduction | | | |  |
| Background/rationale | 2 | Explain the scientific background and rationale for the investigation being reported | 5 | In particular, it is worth investigating the mechanisms where l-apps influence tourist engagement in a tourism-related context rather than a general consumption or retailing context. Such an exploration seems important as it can help tourism app developers improve product quality. |
| Objectives | 3 | State specific objectives, including any prespecified hypotheses | 5-6 | this study aims to integrate users’ digital literacy and their perception of the congruency between digital app presentations and their actual experiences in tourist destinations into the TAM |
| Methods | | | |  |
| Study design | 4 | Present key elements of study design early in the paper | 13-14 | Methods |
| Setting | 5 | Describe the setting, locations, and relevant dates, including periods of recruitment, exposure, follow-up, and data collection | 12 | We randomly invited 400 customers of 71384 users of a tourist center in a central Chinese city through the Internet on Feb, 1st, 2023. |
| Participants | 6 | (*a*) *Cross-sectional study*—Give the eligibility criteria, and the sources and methods of selection of participants | 12 | We randomly invited 400 customers of a tourist center in Northeastern Chinese city through the Internet on Feb, 1st, 2023. The center relies heavily on a location-based consumption app (Dazhongdianping.com) to attract tourists |
|  |  | (*b*) *Cohort study*—For matched studies, give matching criteria and number of exposed and unexposed  *Case-control study*—For matched studies, give matching criteria and the number of controls per case |  |  |
| Variables | 7 | Clearly define all outcomes, exposures, predictors, potential confounders, and effect modifiers. Give diagnostic criteria, if applicable | 18 | Direct effects and indirect effects |
| Data sources/ measurement | 8* | For each variable of interest, give sources of data and details of methods of assessment (measurement). Describe comparability of assessment methods if there is more than one group | 13 | Measures |
| Bias | 9 | Describe any efforts to address potential sources of bias | 15 | Common method bias |
| Study size | 10 | Explain how the study size was arrived at | 12 | We randomly invited 400 customers of 71384 users of a tourist center in a central Chinese city through the Internet on Feb, 1st, 2023. |

| Quantitative variables | 11 | Explain how quantitative variables were handled in the analyses. If applicable, describe which groupings were chosen and why |  |  |
| --- | --- | --- | --- | --- |
| Statistical methods | 12 | (*a*) Describe all statistical methods, including those used to control for confounding | 16, 19 | Table 2. Results of validity and reliability  Table 4. Results of direct effects  Table 5 Result of the indirect effect |
|  |  | (*b*) Describe any methods used to examine subgroups and interactions |  |  |
|  |  | (*c*) Explain how missing data were addressed |  |  |
|  |  | (*d*) *Cohort study*—If applicable, explain how loss to follow-up was addressed  *Case-control study*—If applicable, explain how matching of cases and controls was addressed  *Cross-sectional study*—If applicable, describe analytical methods taking account of sampling strategy |  |  |
|  |  | (*e*) Describe any sensitivity analyses |  |  |
| Results | | | | |
| Participants | 13* | (a) Report numbers of individuals at each stage of study—eg numbers potentially eligible, examined for eligibility, confirmed eligible, included in the study, completing follow-up, and analysed | 12 | We received 346 responses by Feb, 28, 2023; after excluding 17 invalid questionnaires, the sample for analysis consisted of 319 responses, or 92.2% of the total. |
|  |  | (b) Give reasons for non-participation at each stage |  |  |
|  |  | (c) Consider use of a flow diagram |  |  |
| Descriptive data | 14* | (a) Give characteristics of study participants (eg demographic, clinical, social) and information on exposures and potential confounders | 13 | Table 1 Demographic information |
|  |  | (b) Indicate number of participants with missing data for each variable of interest |  |  |
|  |  | (c) *Cohort study*—Summarise follow-up time (eg, average and total amount) |  |  |
| Outcome data | 15* | *Cohort study*—Report numbers of outcome events or summary measures over time |  |  |
|  |  | *Case-control study—*Report numbers in each exposure category, or summary measures of exposure |  |  |
|  |  | *Cross-sectional study—*Report numbers of outcome events or summary measures | 13 | Table 1 Demographic information |
| Main results | 16 | (*a*) Give unadjusted estimates and, if applicable, confounder-adjusted estimates and their precision (eg, 95% confidence interval). Make clear which confounders were adjusted for and why they were included | 17 | Table 3. Results of Correlation and Discrimination Analysis |
|  |  | (*b*) Report category boundaries when continuous variables were categorized |  |  |
|  |  | (*c*) If relevant, consider translating estimates of relative risk into absolute risk for a meaningful time period |  |  |

Continued on next page

| Other analyses | 17 | Report other analyses done—eg analyses of subgroups and interactions, and sensitivity analyses |  |  |
| --- | --- | --- | --- | --- |
| Discussion | | | | |
| Key results | 18 | Summarise key results with reference to study objectives | 21 | Our results suggest that tourists’ digital literacy has a positive impact on their engagement. |
| Limitations | 19 | Discuss limitations of the study, taking into account sources of potential bias or imprecision. Discuss both direction and magnitude of any potential bias | 25 | Limitations and Suggestions for Future Studies: |
| Interpretation | 20 | Give a cautious overall interpretation of results considering objectives, limitations, multiplicity of analyses, results from similar studies, and other relevant evidence | 23 | Theoretical Implications: |
| Generalisability | 21 | Discuss the generalisability (external validity) of the study results | 22 | Our results are generalizable to similar l-apps used by tourists in China. |
| Other information | |  | | |
| Funding | 22 | Give the source of funding and the role of the funders for the present study and, if applicable, for the original study on which the present article is based | 1 | The authors received no financial support for the research, authorship, and/or publication of this article. |

*Give information separately for cases and controls in case-control studies and, if applicable, for exposed and unexposed groups in cohort and cross-sectional studies.

**Note:** An Explanation and Elaboration article discusses each checklist item and gives methodological background and published examples of transparent reporting. The STROBE checklist is best used in conjunction with this article (freely available on the Web sites of PLoS Medicine at http://www.plosmedicine.org/, Annals of Internal Medicine at http://www.annals.org/, and Epidemiology at http://www.epidem.com/). Information on the STROBE Initiative is available at www.strobe-statement.org.
