# Supplementary material for: Enhancing tourist loyalty through location-based service apps: Exploring the roles of digital literacy, perceived ease of use, perceived autonomy, virtual-content congruency, and tourist engagement
Source: PLoS One. 2024 Jan 31;19(1):e0294244. doi: 10.1371/journal.pone.0294244 (PMC10830003; doi:10.1371/journal.pone.0294244)
Supplement: S1 File — (PDF) [file pone.0294244.s003.pdf]

Ethical Approval Form

Researcher Information:

Name: Tong Zhang

Affiliation: The College of Post and Telecommunication of WIT

Email: 92000268@wit.edu.cn

Project Title: Enhancing tourist loyalty through location-based service apps: Exploring the roles of digital literacy, perceived ease of use, perceived autonomy, virtual-content congruency, and tourist engagement.

Research Objectives:

This study will integrate the technology-acceptance model (TAM) into a unique context to examine the roles of digital literacy, perceived ease of use, perceived autonomy, virtual-content congruence, and tourist engagement on tourist loyalty.

Research Methodology:

We will test the hypothesized relationships through structural equation modelling using AMOS 24.0.

Participants:

We will survey the randomly chosen 400 tourists of a local tourist center. The center relies heavily on a location-based consumption app (Dazhongdianping.com) to attract tourists. Those participants should have experience using the app.

Informed Consent:

Explain how you will obtain informed consent from participants, ensuring that they are fully aware of the purpose, procedures, potential risks, benefits, and their rights regarding their participation in the research.

Confidentiality:

In the survey webpage, we will introduce the objective of this study, ensure tourists' voluntary and anonymous participation.

Ethical Considerations:

This project involves neither conflicts of interest, nor any potential harm to participants, cultural sensitivity, or any other ethical issues.

Ethical Approval:

By signing below, I acknowledge that I have read and understood the ethical considerations outlined in this form. I affirm that I will adhere to the ethical principles and guidelines set forth by the Research Ethics Committee and any applicable national or international regulations. I commit to conducting the research with the highest ethical standards and ensuring the welfare and rights of the participants involved.

Researcher's Signature: _Tong Zhang___

Date: __January, 6th, 2023__

武汉工程大学邮电与信息工程学院 艺术设计学院

The College of Post and Telecommunication of WIT
No. 366, Huquan Street, Wuhan, 430073, China

Research Ethics Committee Approval:

The Research Ethics Committee has reviewed the ethical considerations and research methodology presented in this form. After careful assessment, we hereby grant ethical approval for the independent research project described above.

Committee Chair's Name: ____________

Committee Stamp: ______________

Date: _January, 10th, 2023_

The College of Post and Telecommunication of WIT
No. 366, Huquan Street, Wuhan, 430073, China
